# Tools for Molecular Epidemiology of *Streptococcus suis*

**DOI:** 10.3390/pathogens9020081

**Published:** 2020-01-27

**Authors:** Rujirat Hatrongjit, Nahuel Fittipaldi, Marcelo Gottschalk, Anusak Kerdsin

**Affiliations:** 1 Department of General Sciences, Faculty of Science and Engineering, Kasetsart University Chalermphrakiat Sakon Nakhon Province Campus, Sakon Nakhon 47000, Thailand; 2 Public Health Ontario, Toronto Laboratory, Toronto, ON M5G 1M1, Canada; Nahuel.Fittipaldi@oahpp.ca; 3 Department of Laboratory Medicine and Pathobiology, and Department of Cell and Systems Biology, University of Toronto, Toronto, ON M5S 1A1, Canada; 4 Faculty of Veterinary Medicine, University of Montreal, Montreal, QC H3T 1J4, Canada; marcelo.gottschalk@umontreal.ca; 5 Faculty of Public Health, Kasetsart University Chalermphrakiat Sakon Nakhon Province Campus, Sakon Nakhon 47000, Thailand

**Keywords:** *Streptococcus suis*, clonal complex, multilocus sequence typing (MLST), PCR, minimum core genome sequence typing (MCG), pathotyping

## Abstract

Diseases caused by *Streptococcus suis* are a significant economic and welfare concern in pigs as well as in humans. Several molecular methods have been applied to investigate *S. suis* strain diversity and identify phylogenetic groups. Multilocus sequence typing (MLST), commonly used to differentiate between *S. suis* strains, has been instrumental in identifying that the species is genetically highly diverse. Recent advances in whole-genome analysis have resulted in schemes permitting the classification of *S. suis* populations as pathogenic or non-pathogenic, or disease-associated or non-disease associated. Here, we review these and other molecular approaches that can be used for surveillance, outbreak tracking, preventative health management, effective treatment and control, as well as vaccine development, including PCR based-assays that are easy to apply in modest diagnostic settings and which allow for the rapid screening of a large number of isolates at relatively low cost, granting the identification of several major clonal complexes of the *S. suis* population.

## 1. Introduction

*Streptococcus suis* is a pathogen of pigs that can cause infections in humans who are in close contact with infected animals and/or contaminated pork-derived products, as well as those who have consumed raw pork products [1]. *S. suis* can cause meningitis, septicemia, endocarditis, and arthritis in humans [2,3,4]. Human *S. suis* cases have been reported in most countries of Western Europe, North and South America, Africa, Australia, New Zealand, India, Japan, and several other East and Southeast Asian countries, particularly China, Vietnam, and Thailand [1,3,4]. Indeed, *S. suis* has been shown to be responsible for thousands of human cases, of which 90.2% were in Asia, 8.5% in Europe, and 1.3% in other parts of the world [4].

Previously, *S. suis* had been classified into 35 serotypes (serotype 1/2, and 1–34) and which were then reduced to 33 serotypes because serotypes 32 and 34 were re-classified as *S. orisratti* [5,6,7,8,9]. In 2013, *S. suis* serotypes 20, 22, 26, and 33 were proposed to be removed from the *S. suis* taxon [10]: serotypes 20, 22, and 26 were proposed as *Streptococcus parasuis* [11,12], while serotype 33 was classified as *Streptococcus ruminantium* [13]. Hence, currently there are 29 true *S. suis* serotypes. Of them, serotype 2 is the most prevalent in both human and pig infections although cases caused by serotypes 4, 5, 9, 14, 16, 21, 24, and 31 have also been reported [1,2,3,4,14,15,16,17].

Studying the population structure and the genetic diversity of *S. suis* is helpful to understand the epidemiology of this organism as well as reveal clones or clonal groups with an apparently increased capacity to cause disease, or which are potentially associated with particular clinical manifestations. Several molecular typing techniques have been applied to the study of *S. suis* genetic diversity (Table 1); for example, pulse-field gel electrophoresis (PFGE) [18,19], randomly amplified polymorphic DNA (RAPD) [20,21], amplified fragment length polymorphism (AFLP) [22], polymerase chain reaction-restriction fragment length polymorphism (PCR-RFLP) [23], ribotyping [24,25], multilocus sequence typing (MLST) [26], and multilocus variable number tandem repeat analysis (MLVA) [27].

Of these techniques, MLST is a popular method that has been used throughout the world to determine the sequence types (STs) as well as clonal complexes (CCs) of *S. suis* strains isolated from pigs and humans, which has enhanced our understanding of the global *S. suis* epidemiology. More recently, the use of whole-genome sequencing (WGS; Table 1) has been instrumental in defining *S. suis* isolates as being members of epidemic/highly virulent (E/HV), virulent (V), or intermediately/weakly virulent (I/WV) groups [28]. Bayesian Analysis of Population Structure group (BAPS) [29] and minimum core genome group (MCG) analyses have also been used to characterize *S. suis* isolates [30,31]. Recently a pathotyping tool has been proposed to differentiate between disease-associated and non-disease associated groups [32]. Here, we review these current methods which have been applied to determine the population structure of *S. suis* isolates.

## 2. Multilocus Sequence Typing (MLST)

MLST is considered the gold standard to determine the structure of *S. suis* populations. This powerful method uses genetic variation that accumulates very slowly in housekeeping genes to investigate the genetic diversity of *S. suis* and its use has allowed global and long-term epidemiology. King et al. established the MLST scheme for *S. suis* in 2002, using seven different house-keeping genes (*cpn60*, *dpr*, *recA*, *aroA*, *thrA*, *gki*, and *mutS*) [26]. Since its establishment, this MLST scheme has been adopted by multiple laboratories throughout the world and used to determine the STs of *S. suis* strains isolated from pig and human cases of infection [1].

**Figure 1:**
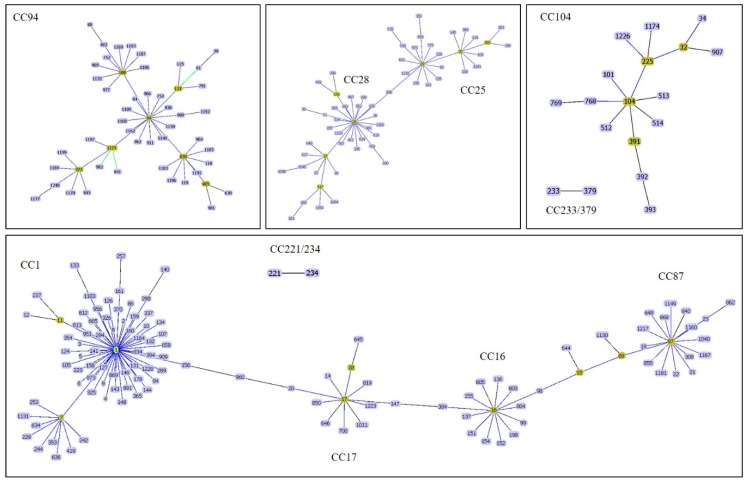
A goeBURST analysis of major clonal complexes of *S. suis* causes of infections in human and pig. CC1 is related to CC17 whereas CC17 is closely related to CC16. CC16 and CC87 are related via ST15 and ST89. CC25 and CC28 are related via ST856. Other CCs are independent.

Thus far (28 November 2019), a total of 1245 STs have been recorded in the *S. suis* MLST database (https://pubmlst.org/ssuis/). This method has revealed the presence of many clonal complexes (CCs) within the *S. suis* population in the database. Among the different CCs identified, the most important causes of infections in human and pig have been CC1, CC16, CC20, CC25, CC28, CC94, CC104, CC233/379, and CC221/234 (Figure 1) [1,4,17,33]. Different CCs were found to be distributed in different regions of the world [1]. CC1 was found mostly in Europe, Asia (Cambodia, China, Korea, Japan, Thailand, and Vietnam), Australia, and South America (Argentina), while CC20 (ST20) was described as being important in the Netherlands [1,33,34,35,36,37]. Furthermore, ST7 (CC1), responsible for the 1998 and 2005 epidemics, was mostly present in China [1] and CC16 and CC94 were predominant in Europe, although human cases were reported in Thailand [17,33]. CC25 or CC28 were reported in North America and were also recovered in Thailand, Korea, Japan, and Australia [1,4,33,34,35,36,37]. Finally, CC104 (ST101, ST104, ST391-ST393, ST512-ST514), and CC233/379 (ST233 and ST379) were endemic to Thailand [1,33]. In North America, the structure of *S. suis* strains is more complicated and pathotypes are different from Europe and Asia [38]. Serotypes 1, 1/2, 2, 7, 14, and 23 as well as ST1, ST13, ST28, ST94, ST108, ST961, and ST977 have recently been described as pathogenic strains in the USA [38].

An alternative MLST approach using matrix-assisted laser desorption ionization-time of flight mass spectrometry (MS-MLST) has been reported to be more rapid in providing typing results than dideoxy sequencing [36]. While both MS-MLST and conventional MLST had 100% concordance in their classification of sequence types (STs), in addition to faster time-to-results, MS-MLST had lower labor requirements and per-isolate costs. MS-MLST analysis was easier to instrument when large numbers of isolates were involved [36]. However, MS-MLST requires a high initial investment in the MALDI-TOF MS equipment and software. Therefore, this approach may not be readily available to most laboratories, particularly those in developing countries.

## 3. Prediction of CC Using Polymerase Chain Reaction

Although informative, MLST is a high-cost and time-consuming method that is not ideal for screening large numbers of isolates. MLST is also unavailable in many laboratories in developing countries. Therefore, polymerase chain reaction (PCR)-based approaches [38,39,40,41,42] to identify the most important *S. suis* CCs (PCR-CC) were developed allowing for the rapid screening of a large number of isolates at a relatively modest cost (summarized in Table 2).

A first study reported that a variant of the serum opacity factor (*ofs*) can serve as a genetic marker to determine CCs of *S. suis* [39]. The report showed that a type-1 *ofs* was strongly associated with CC1, while type-3 *ofs* and type-4 *ofs* were associated with CC27. Importantly, CC25, CC28, and CC104 were included in CC27 with the less-stringent group definition (5 alleles sharing) used in that study [39]. However, since type-3 *ofs* was present in both CC94 and CC27, these two CCs could not be resolved. Similarly, CC25 and CC104 could not be differentiated from CC27.

A second study described a PCR assay to determine how pilus-associated gene profiles correlated with CCs of *S. suis* [40]. The results showed that such gene profiling could be used as a screening method for potentially hazardous *S. suis* groups. For example, genotype A of the pilus-associated gene profile was strongly associated with CC1, genotype B was associated with CC25 and CC27, and genotype C was related to CC104. Despite its enhanced discriminatory power, this assay cannot distinguish between CC25 and CC27, and multiple PCR reactions are necessary to identify the gene profiles for each genotype.

A third study reported a multiplex PCR that can be used to identify *S. suis* CCs that are relevant to human infections in a single reaction [41]. Of the 7 sequences encoded—2 hypothetical proteins (*hp1* and *hp2*), 1 ribonuclease G (*ribG*), 1 peptidase (*pep*), 1 muramidase-like protein (*mp*), 1 zonula occludens toxin (*zot*), and 1 collagen adhesion (*col*)—only *hp1*, *mp*, *pep*, and *col* were selected as targets for PCR with the srtBCD primers described by Takamatsu et al. (2009). The test developed in that study differentiated between CC1, CC25, CC28, CC104, CC221/234, and CC233/379. However, the assay could not detect CC16, CC20, and CC94, and the report did not evaluate assays on CCs mostly related to pig infections.

A fourth report by Maneerat et al. (2013) demonstrated that RAPD using OPB-07, OPB-10, and OPB-17 could distinguish CC1, CC104, CC233/379, and ST336 [42]. This study revealed a high agreement of RAPD patterns among the three primers used with the specific pattern of each CC in each primer. Nonetheless, this study did not evaluate other *S. suis* CCs, such as CC25, CC28, and CC16.

A last report used random amplification of polymorphic DNA (RAPD) and 16S-23S rDNA intergenic spacer polymerase chain reaction-restriction fragment length polymorphism (16S-23S PCR-RFLP) to determine clonal complexes (CCs) of 684 *S. suis* isolates from pigs (n=72) and humans (n=612) [43]. The study also revealed that RAPD was better at distinguishing the major *S. suis* CCs than the PCR-RFLP method: the assay was capable of simultaneously distinguishing CC1, CC16, CC25, CC28, CC104, CC221/234, and CC233/379. RAPD with the OPB-10 primer clearly distinguished each CC in the study, and no cross-patterns were found. Finally, the study also showed that neither the OPB-06 nor OPB-11 primer could distinguish CC104 from CC233/379 isolates. The report showed that PCR-RFLP and DNA sequencing using the 16S-23S rDNA intergenic spacer could not clearly differentiate among most CCs [43]. Both techniques could classify *S. suis* isolates into four clusters: cluster 1 consisting of CC25, CC28, CC104, and CC233/379; cluster 2 consisting of CC221/234; cluster 3 consisting of CC16 (ST16); and cluster 4 consisting of CC1.

It is important to note that pig *S. suis* isolates are likely to be much more diverse than zoonotic human isolates. However, none of abovementioned studies evaluated *S. suis* CCs related to pig infections such as CC7, CC11, CC13/149, CC43/52, CC53/54, CC87, CC94, CC123, CC147, CC226/227, CC239/241, and CC20.

In addition to these PCR-based techniques, MLVA is a technique with high discriminatory power that has also been used to investigate *S. suis*. While PFGE could not differentiate between isolates, one MLVA scheme using nine loci (TR1-TR9) was successfully used to link and to sub-type *S. suis* ST7 isolates from two outbreaks that occurred in China in 1998 and 2005 [27]. Additional advantages of MLVA are that it is relatively easy to perform, rapid and reliable, and that it permits high-throughput screenings. MLVA also allows the comparison of results between laboratories and is useful in case of outbreak investigations.

## 4. Whole-Genome Sequencing Approaches

WGS approaches have increasingly been used to investigate *S. suis* isolates, including molecular determination of serotype [44], characterization of outbreaks [45,46], evaluation of *S. suis* reinfection [47], and to determine the population structure of *S. suis* isolates of serotype 2 belonging to ST25 and ST28 [48,49], as well as of serotype 9 [50]. WGS-based bacterial typing strategies commonly use one of two approaches: SNP (sequence)-based, or MLST (allele)-based. The SNP approach compares single nucleotide differences between isolates in comparison to a reference genome and is particularly useful to determine the clonal relationship between highly similar isolates. The MLST approach is an extension of conventional 7-gene MLST that expands the range of genes to the genome scale and can be roughly divided into core-genome-based MLST (cgMLST) and whole-genome-based MLST (wgMLST). Allele-based comparisons can be conducted using analysis tools available on the internet, such as BacWGSTdb (http://bacdb.org/BacWGSTdb/) [51], pubMLST (https://pubmlst.org/ssuis/), or the Center for Genomic Epidemiology (https://cge.cbs.dtu.dk/services/MLST/) [52].

The definition of virulence in *S. suis* remains controversial, and WGS-based approaches have played an important role in the efforts to identify virulence markers permitting to differentiate between commensal organisms and those prone to cause disease. A comparative genome hybridization (CGH) was used to analyze 55 *S. suis* isolates from different serotypes, recovered from different hosts, different clinical sources, and different geographical locations [53]. Clustering of CGH data divided *S. suis* isolates into 2 clusters. Cluster A exclusively contained virulent CC1 isolates of serotypes 1 and 2 isolates [53]. Cluster B, on the other hand, contained mainly a more divergent and heterogeneous group of serotype 7 and 9 isolates [53]. Another study used CGH on 39 isolates of different serotypes, sources, geographic locations, isolation years, and STs. This study revealed that the *S. suis* strains tested could be classified into three groups of differing levels of virulence: (i) epidemic and highly virulent (E/HV group), which included ST1, ST6, ST7, and ST11 isolates; (ii) virulent (V group), containing ST81, ST13, ST56, ST87, ST308, ST54, and ST53 isolates; and (iii) intermediately or weakly virulent (I/WV group), composed of isolates belonging to several STs that were all recovered from non-human sources [28].

The genomes of 375 *S. suis* isolates with detailed clinical phenotypes from pigs and humans from the United Kingdom and Vietnam were analyzed using a Bayesian Analysis of Population Structure (BAPS) [54]. The study showed clear genetic differences between systemic, respiratory, and non-clinical (carriage) *S. suis* isolates. Interestingly, systemic isolates had a smaller genome than respiratory and carriage isolates, and they tended to have an over-representation of virulence factors and a larger number of genes involved in defense functions [54]. Willemse et al. (2016) used BAPS to investigate the whole genomes of 98 *S. suis* isolates from human patients and pigs with invasive disease in the Netherlands, and 18 genomic complete and available *S. suis* sequences. The BAPS-based population grouping did not correlate with serotype but correlated well with the CC of the isolates. BAPS Group 1 comprised all CC13 isolates, BAPS Group 2 comprised most of the CC16 isolates, BAPS Group 4 comprised all CC1 isolates, BAPS Group 5 comprised both the CC27 and the CC29 isolates, and BAPS Group 6 comprised most CC20 isolates [29].

Minimum core genome sequence typing (MCG) was proposed by Chen et al. in 2013 [30]. These authors sequenced the genomes of 85 *S. suis* isolates belonging to 75 STs and defined an MCG of 876 genes and 58,501 SNPs. Using this variation, they reported seven MCG groups among their strain population. MCG Group 1 included all the highly virulent isolates of ST1 and the epidemic isolates of ST7, as well as all isolates from human infections and outbreaks, while the intermediate virulent STs, such as ST25 and ST28, were located in MCG Group 4 [30]. As MCG typing using whole-genome sequencing is unavailable and not cost effective in limited-resource countries, a single nucleotide polymorphisms-based typing method using PCR amplification and sequencing of six genes covering the distribution of 10 SNPs has been developed to identify the seven MCG groups [31]. This approach can categorize isolates into the correct MCG groups and offers a practical, rapid, and cost-effective assay for differentiating the population structure of *S. suis.*

Very recently, a genome-wide association study that took into consideration the observed clinical phenotype (invasive and asymptomatic) and used the discriminant analysis of principal components (DAPC model) implemented in the R package *adegenet* was conducted, which identified genetic markers of invasive disease and asymptomatic tonsillar carriage [32]. The identified markers were used to develop a pathotyping tool able to predict the virulence potential of *S. suis* isolates as disease-associated and non-disease associated [32] and also led to the development of a multiplex PCR assay for the identification of three *S. suis* genetic-markers that differentiated 115 *S. suis* isolates into disease-associated and non-disease-associated groups. The multiplex PCR assay revealed a good correlation compared to the observed clinical phenotypes, serotyping, virulence-associated gene (*epf*, *mrp*, and *sly*) profile, MLST, and MCG sequence type [32].

High-throughput sequencing systems are now available at many public health and hospital diagnostic laboratories, and the use of these systems in rapid diagnosis of *S. suis* has shown great potential. A report from Canada described that WGS (Illumina MiSeq) was instrumental in identifying *S. suis* serotype 2 ST25 as the causative agent of a human infection in a farmer, which had previously been ascribed to the viridans group *Streptococcus* by biochemical methods [55]. More recently, Hayashi et al. (2019) used Illumina short-reads to investigate two human *S. suis* cases in Japan. The genomic approach revealed that the isolates belonged to serotype 2 ST28, were derived from the same genetic lineage, and were closely related to *S. suis* strains previously isolated from pigs in that country [56]. The Oxford Nanopore MinION sequencer has also been used to rapidly generate a full-length *S. suis* consensus genome sequence, achieving a 99.4% average accuracy. Using optimized methods, this technology also permitted to determine the antibiotic resistance profile and sequence type of the isolates, with additional error corrections [57]. This report could build towards a standardization of the WGS workflow, for routine and low-cost disease diagnostics that are easy to perform, as well as have a short turnaround time.

## 5. Conclusions

Several methods exist to investigate strain diversity and identify phylogenetic groups of *S. suis*. Serotyping and MLST are still commonly used to differentiate *S. suis* strains. Advances in sequencing technologies, such as WGS, have allowed the determination of the population structure of *S. suis* strains and identified groups differing in virulence potential using Bayesian analysis, minimum core genome analysis, or genome-wide association. Collectively, PCR assays, which are easy to apply in routine diagnostic testing, permit the identification of several major groups of the *S. suis* population. Molecular tools could be used for surveillance and preventative health management through the detection of clones or strains that have genetic potential to cause disease in healthy animals. Furthermore, these techniques could be applied to track isolates in an outbreak, select strains for a vaccine, and develop effective treatment and control plans.

## Figures and Tables

**Table 1:** Characteristics of the molecular epidemiological methods for the *Streptococcus suis* study.

Characteristic	WGS	MLST	Multiplex PCR-CC	RAPD	PCR-RFLP	MLVA	AFLP	PFGE	Ribotyping
Reproducibility	Good	Good	Good	Poor to moderate	Moderate	Good	Good	Good	Good
Discriminatory power	Excellent	High	Moderate	Moderate to good	Poor to moderate	Excellent	Excellent	Excellent	Good
Ease of use	Moderately labor-intensive	Simple to moderate labor	Simple	Simple	Simple	Simple	Moderate	Labor-intensive	Labor-intensive
Interpretation	Moderate to very complex	Simple to moderate	Simple	Moderate to complex	Simple	Simple	Complex	Moderate to complex	Moderate to complex
Cost	Very high	Moderate	Low	Low	Low	Low to moderate	Moderate	High	High
Universal applicability	Yes	Yes	Limit to some CCs	Yes	Yes	Yes	Yes	Yes	Yes

**Table 2:** PCR-predicted important clonal complexes of *S. suis* isolates.

Clonal Complexes	PCR Methods				
	Multiplex PCR	PCR of *ofs* Genes	PCR-Pilus-Associated Gene Profiles	RAPD	16S-23S rDNA PCR-RFLP
CC1	🗸	🗸	🗸	🗸	🗸
CC16	🗶	🗶	🗶	🗸	🗸
CC20 **	🗶	🗶	🗶	🗶	🗶
CC25	🗸	🗸 *	🗸 *	🗸	🗸 *
CC28	🗸	🗸 *	🗸 *	🗸	🗸 *
CC94	🗶	🗸 *	🗶	🗶	🗶
CC104	🗸	🗸 *	🗸	🗸	🗸 *
CC233/379	🗸	🗶	🗶	🗸	🗸 *
CC221/234	🗸	🗶	🗶	🗸	🗸

Note: * reveal the same profile; thus, could not be differentiated for each. ** PCR methods were not applied to CC20.
